# Catarrh of the Uterus

**Published:** 1870-10

**Authors:** 


Catarrh of the Uterus.—Dr. G. W. Hamilton, writing on this disease in the *Buffalo Med. and Surgical Journal*, claims that its pathology consists in dilation of the capillaries and congestion of the lining of the uterus. When there is no ulceration, he advocates the use—in addition to good hygiene, and such medicines as will regulate the bowels and secretions, and improve the digestion and general health—of injections of persulphate of iron, of the strength of 24 grs. to the half-pint of water, and the internal use of ergot in moderate doses for several weeks continuously. The ergot operates by increasing the tonicity of the capillary bloodvessels of the uterus, improving the circulation and removing the congestion. Ergot does not retard the circulation unless given continuously and in large doses; it may be given a long time in proper medicinal doses, without any bad effect whatever.

